# Cell Membrane Coated-Biomimetic Nanoplatforms Toward Cancer Theranostics

**DOI:** 10.3389/fbioe.2020.00371

**Published:** 2020-04-29

**Authors:** Tingting Li, Xiang Qin, Yichao Li, Xue Shen, Shun Li, Hong Yang, Chunhui Wu, Chuan Zheng, Jie Zhu, Fengming You, Yiyao Liu

**Affiliations:** ^1^Department of Biophysics, School of Life Sciences and Technology, University of Electronic Science and Technology of China, Chengdu, China; ^2^Center for Information in Biology, University of Electronic Science and Technology of China, Chengdu, China; ^3^Sichuan Industrial Institute of Antibiotics, Chengdu University, Chengdu, China; ^4^Department of Cancer Research, Hospital of Chengdu University of Traditional Chinese Medicine, Chengdu, China

**Keywords:** cancer, theranostic, cell membrane, biomimetic, nanocomplex

## Abstract

Research of nanotechnology for cancer therapy and diagnosis extends beyond drug delivery into the targeted site or surveillance the distribution of nanodrugs *in vivo* or distinction tumor tissue from normal tissue. To satisfy the clinic needs, nanotheranostic platform should hide the surveillance by immune system and the sequestration by filtration organs (i.e., liver and spleen). Use of biologically derived cellular components in the fabrication of nanoparticles can hide these barriers. In this review, we update the recent progress on cell membrane-coated nanoparticles for cancer theranostics. We hope this review paper can inspire further innovations in biomimetic nanomedicine.

## Introduction

Cancer is known to be the most aggressive malignancy to humans, and definitely the major cause of death worldwide (Roma-Rodrigues et al., 2019). In the fight against cancer, half of the battle is won based on its early detection (Phillips et al., 2014; Ye et al., 2014). As we know, new treatments such as phototherapy and immunotherapy have received considerable attention due to their obvious advantages than the conventional therapies. Phototherapy including photothermal therapy (PTT) and photodynamic therapy (PDT), they rely on the phototherapeutic agents in cooperation with laser irradiation to selectively kill cancer cells, while ignoring the healthy cells in the dark (Liu X. et al., 2018; Li W. et al., 2019). However, monotherapies have limited efficacy. Consequently, multiple approaches have been provided to be a promising route for cancer therapy (Min et al., 2019; Yue et al., 2019).

Theranostics based on nanotechnology strategies is a new form of cancer treatment, they can integrate the conventional or the new therapeutic modalities and diagnostic functions such as magnetic resonance (MR) imaging, photoacoustic (PA) imaging, positron emission tomography (PET) imaging or fluorescence imaging etc. into one single carrier and assist in the management of cancer (Li et al., 2016; Li T. et al., 2019). Theranostics showed a number of advantages such as improved diagnosis, tumor specific delivery of drugs, reduced damage to healthy tissue.

In the last few decades, theranostic nanoplatforms gained great progress in basic research and produced a large number of excellent publications (Yang H. et al., 2018; Shen et al., 2019). Scientists developed numerous theranostic nanosystems based on organic nanoparticles, inorganic nanoparticles, micelles, dendrimer, and got good effect in cell studies and animal studies (Ray et al., 2018; Yang J. et al., 2018; Shao et al., 2019; Xu et al., 2019; Bort et al., 2020). These theranostic nanoplatforms were modified with polymer, antibody, peptide or other functional molecules to obtain profuse biological functions, such as targeting, long circulation, biocompatibility, and immune escaping (Yang et al., 2017; Liu S. et al., 2019; Xie X. et al., 2019; Wu et al., 2020).

But the application of theranostic nanoplatforms in the clinical trials have been disappointing. Two vital problems need to be solved urgently, the first one is the biosafety of theranostic platforms should be systemically evaluated. The second one, which our final goal, is getting the expected effect in the clinic (Choi et al., 2012; Liu Y. et al., 2018; Vankayala and Hwang, 2018; Jing et al., 2019). As a consequence, we should develop theranostic nanosystems that closely mimic the biological composition of our bodies and make the efficiency of theranostic nanoplatforms maximally.

**FIGURE 1:**
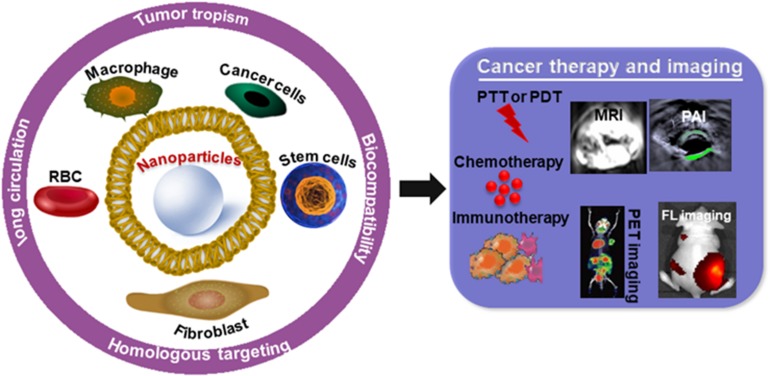
The application of cell membrane-coated nanoparticles for cancer therapy and imaging.

Cell membrane coated biomimetic nanoplatforms are often semi-biological (or semi-artificial) which take advantages of their inherited property, such as biointerfacing, self-identification and signal transduction can escape from biological barriers such as immune clearance, opsonization, and negotiation with vascular system (Evangelopoulos and Tasciotti, 2017; Chen et al., 2019; Jin et al., 2019; Madamsetty et al., 2019; Yan et al., 2019; Ma et al., 2020a). These theranostic nanoplatforms have the potential to play an important role in cancer diagnosis and treatment (Bose et al., 2018a; Li Z. et al., 2018; Meng et al., 2018; Sung et al., 2019; Ye S. et al., 2019). This review article will introduce the recent efforts on the rational design of cell membrane-based biomimetic nanosystem for cancer diagnosis and treatment, we highlight the strategies of engineering and application in Figure 1.

## Development of Cell Membrane-Coated Biomimetic Theranostic Nanoplatform

Hu et al. (2011) first reported that they used a top-down method to synthesize erythrocyte membrane camouflaged nanoparticles for long-circulating cargo delivery. Since that, cell membrane, not just erythrocyte membrane but cancer cell membrane, stem cell membrane, platelet membrane, endothelial cell membrane, etc. were used for coating materials of nanoparticles (Kroll et al., 2017; Narain et al., 2017; Pasto et al., 2019; Zhou et al., 2019). Owning the advantages of the native functionalities originating from cell membrane including reserved antigens and cell membrane structure, biomimetic nanoparticles can acquire special functions, such as ligand recognition and targeting, long blood circulation, and immune escaping (Chen Z. et al., 2016; Feng et al., 2019; Meng et al., 2019). In a valuable review paper, the authors discussed in detail the advantages of different cell membrane camouflaged nanoparticles (Chen Z. et al., 2016). In this paper, we briefly summarized the recent progress in the development of biomimetic cell membrane camouflaged nanocomplex for cancer theranostic. We also demonstrated the highlight in the recent research about biomimetic cell membrane camouflaged nanocomplex on cancer theranostic in the following sections.

## Red Blood Cells Membrane Camouflaged Theranostic Nanocomplex

**FIGURE 2:**
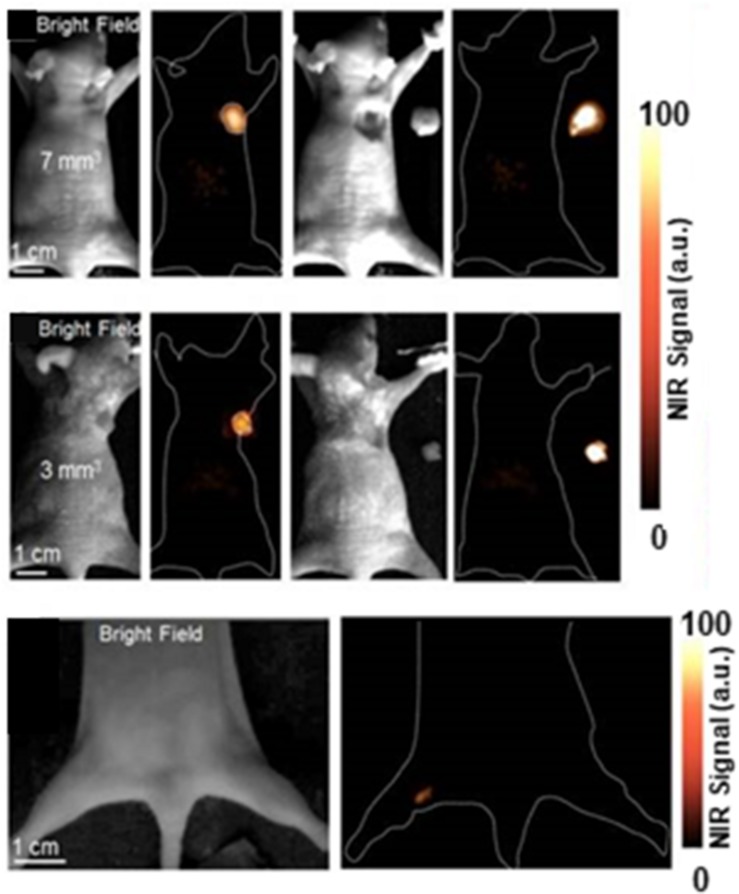
NIR-II fluorescence imaging guided tumor surgery of RBCp. NIR-II fluorescence bioimaging results of epidermal tumors with sizes of 7 mm^3^ (row one), 3 mm^3^ (row two), and the NIR-II bioimaging results of popliteal lymph node metastasis (row three). Reproduced with permission from Wang et al. (2019). Copyright © Ivyspring International Publisher.

Red blood cells (RBCs) are the primary transport of oxygen through the blood in body, they can live up to 120 days in humans, and are nature’s long circulating carriers (Hsieh et al., 2015; Fang et al., 2018; Liu J.M. et al., 2018). It is reported that RBCs are the ideal membrane modification materials because of the abundant proteins, glycans, and receptors on the RBCs membrane surfaces which can bypass the immune system attack (Rao et al., 2017; Ren et al., 2017; Su et al., 2017). For example, CD47 (integrin-associated protein) is a self- marker of RBCs which can interface with its corresponding receptor, prevent the clearing from the bloodstream by macrophages (Oldenborg et al., 2000; Xie J. et al., 2019). The intact RBCs membrane could directly modify on the surface of nanoparticles without any complex process, and the final nanoparticle still inherits the functions of RBCs (Jiang et al., 2017; Lian et al., 2019; Yang et al., 2019). In one instance, Li et al. used Ag_2_S quantum dots (QD), a good fluorescence imaging agent with ideal photothermal and photodynamic therapeutic effects under laser irradiation as a sonosensitizer. Pluronic F-127-modified Ag_2_S QDs were wrapped in RBC vesicles for enzyme-augmented sonodynamic therapy (SDT). RBC membranes coating in this system could prolong the circulation time of the probe, and catalyzed endogenous H_2_O_2_ by the catalase in RBCs to ameliorate tumor hypoxia. Besides, Ultrasound (US) could also promote tumor blood flow, relieve the hypoxic condition, and enhance the SDT effect of the probe. This study provide a promising strategy for the future design of a multifunctional theranostic nanoplatform (Li et al., 2020). Wang and co-workers designed RBC based probe (RBCp) for NIR-II fluorescence bioimaging-guided tumor surgery and light-triggered O_2_ release to enhance PDT efficiency. *In vivo* study showed that RBCp could provide efficient tumor targeting and laser-responsive O_2_ release to enhance the PDT efficiency of popliteal lymph node metastasis under the guidance of NIR II fluorescence bioimaging (Figure 2; Wang et al., 2019).

## Cancer Cell Membrane Camouflaged Theranostic Nanocomplex

**FIGURE 3:**
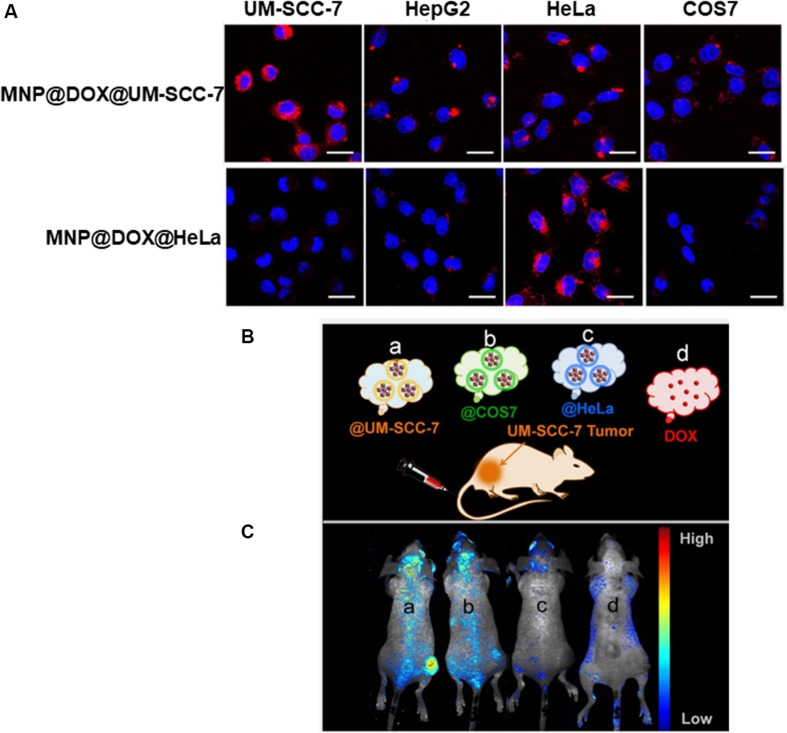
**(A)** Confocal laser scanning microscope (CLSM) images of four cell lines including UM-SCC-7, HepG2, HeLa, and COS7 cells upon 2 h coincubation with MNP@DOX@UM-SCC-7 and MNP@DOX@HeLa. Scale bars: 20 μm. **(B)** Schematic illustration of UM-SCC-7 tumor-bearing mouse model treated with DOX and various cell membrane cloaked MNP@DOX@CCCM. **(C)** *In vivo* fluorescence images at 24 h post intravenous injection with MNP@DOX@CCCM (**a:** @UM-SCC-7; **b:** @COS7; **c:** @HeLa) and DOX **(d)** with an equivalent DOX dosage (2.5 mg/kg). Reproduced with permission from Zhu et al. (2016). Copyright © 2016 American Chemical Society.

Inspired by the reality that nanotheranostic nanoplatform should have good biocompatibility and the ability of homologous targeting, cancer cell membrane coated nanotheranostic nanoplatform have been recently getting more and more attention (Li S.Y. et al., 2018; Zhang N. et al., 2018; Zhang W. et al., 2019; Kumar et al., 2019). In particular, cancer cells are robust and easy to multiply culture *in vitro* for mass membrane collection, cancer cell membrane expressing “markers of self” and “self-recognition molecules” can be removed from cancer cells and coated on nanoparticles, demonstrating homologous targeting and immune escape ability (Bose et al., 2018b; Shao et al., 2018; Cai et al., 2019; Harris et al., 2019; Liu C. et al., 2019; Nie et al., 2019; Zhang D. et al., 2019). Wang and coauthors designed HeLa cell membrane coated nanocomposites for Fluorescence/MR dual-modal imaging guided PDT. These HeLa membrane coated nanocomposites (denoted as mGZNs) showed enhanced *in vivo* anti-tumor targeting efficiency of 80.6% for HeLa cells, providing new strategies to develop nanocomposites for visualized cancer theranostics (Wang et al., 2020). Zhu and coworkers designed a magnetic iron oxide based nanosystem coated with different types of cracked cancer cell membranes (CCCM). This nanocomplex showed the excellent self-recognition internalization by the source cancer cell lines *in vitro* and *in vivo*. As shown in Figure 3A, cellular internalization of UM-SCC-7, and HeLa cell membrane coated MNP@DOX@NPs (termed as MNP@DOX@UM-SCC-7 and MNP@DOX@HeLa, respectively) was studied upon 3 h coincubation with four cell lines including UM-SCC-7, HeLa, HepG2, and COS7 cells. An amazing outcome was found that the fluorescence intensity originating from two CCCM coated nanoparticles was far superior in the corresponding source cells over those in heterotypic cells. To conform the *in vivo* tumor self-targeting ability toward homologous tumors, the authors intravenously injected mice bearing UM-SCC-7 tumor on the right hind limb with MNP@DOX@CCCM NPs prepared with different cell membranes. As shown in Figure 3B, In the group injected with MNP@DOX@UM-SCC-7 showed more intratumor fluorescence intensity than other groups (Zhu et al., 2016).

## Stem Cell Membrane Camouflaged Theranostic Nanocomplex

Stem cell membrane is another natural biomimetic membrane coating that have been used for cancer theranostics (Ma et al., 2019; Shin et al., 2019; Zhao et al., 2019). Stem cell membrane with its inherent tumortropism coating onto nanoparticles has enabled the fabrication of nanocarriers with similar targeting functionality (Letko Khait et al., 2019; Wu et al., 2019). In an example of umbilical cord-derived mesenchymal stem cell coated polymeric nanoplatform, poly(lactic-co-glycolic acid) (PLGA) nanoparticle loaded with Doxorubicin (NP-Dox) were coated with cord-derived mesenchymal stem cell membrane for tumor-targeted delivery of chemotherapy. The coating membrane significantly enhanced the cellular uptake efficiency of PLGA nanoparticles and the tumor cell killing efficacy of PLGA-encapsulated doxorubicin (Yang N. et al., 2018). In another study, bone marrow derived mesenchymal stem cell membrane was coated on gelatin nanogels (termed as SCMGs) for tumor-targeted drug delivery. SCMGs showed high cancer cellular uptake of DOX compared with gelatin-DOX and free DOX. To monitor the *in vivo* distribution of nanogels, a near-infrared fluorescent dye, Cyanine7 (Cy7) was loaded into both SCMGs and bare gelatin nanogels. After intravenous injection of different nanogel formulations in tumor bearing mice, the average fluorescence signal in the SCMGs treated mice was notably higher than that obtained in the group treated with bare gelatin (Gao et al., 2016).

## Cancer-Associated Fibroblast Membrane Camouflaged Theranostic Nanocomplex

**FIGURE 4:**
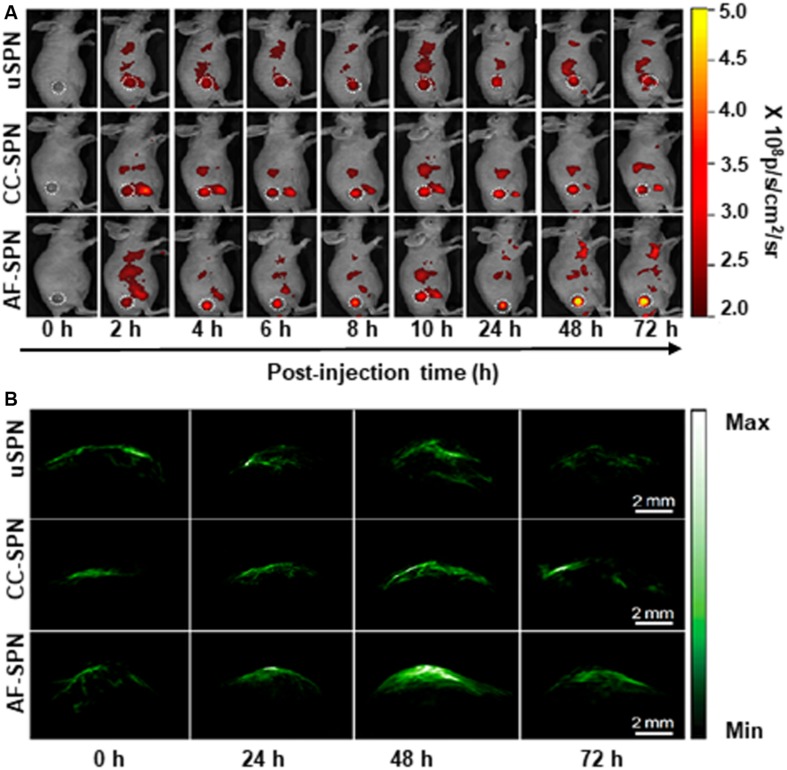
*In vivo* fluorescence and PA imaging of tumors in living mice after treatment with different complexes. **(A)** *In vivo* NIR fluorescence images of 4T1 tumor-bearing mice at different time points after systemic administration of uSPN, CC-SPN, and AF-SPN via tail vein injection. The tumor was on the right side of the back, as indicated by the white dashed circles. **(B)** Representative PA maximum imaging projection of 4T1 tumors in living mice at 0, 24, 48, and 72 h after systemic administration of different complexes via tail vein injection. Reproduced with permission from Li J. et al. (2018). Copyright © 2018 American Chemical Society.

Cancer associated fibroblast membrane has recently obtained more and more attention as membrane coating materials. As reported, cancer-associated fibroblasts are recognized as a key obstacle to cancer treatment (Chen B. et al., 2016). On the one hand, they construct a protecting physical barrier to impede tumor cells uptake of antitumor drugs. One the other hand, they secrete abundant growth factors and cytokines to activate correlative signaling pathways for promoting tumor angiogenesis, progression, initiation, metastasis, and resistance. Studies have shown that used cancer-associated fibroblast membrane coated nanoparticles to deliver therapeutic agents could target and kill cancer-associated fibroblasts, and depleted tumor-stroma biological interactions and in turn led to enhanced therapy (Ji et al., 2015, 2016; Li L. et al., 2018; Kovacs et al., 2020). Li et al. developed semiconducting polymer nanoparticles (SPNs) coated with activated fibroblast membranes (denoted as AF-SPN) for enhanced multimodal cancer phototheranostics. In this study, uncoated SPN (uSPN) nanoparticles and the cancer cell membrane coated SPN (CCSPN) nanoparticles were as control group. To demonstrate the photodiagnostic potential of nanoparticles *in vivo*, different nanoparticles were intravenously injected into 4T1 tumor-bearing mice, and their NIR fluorescence and PA images were obtained. As shown in Figures 4A,B. AF-SPN facilitated homologous targeting ability, and allowed to specifically target cancer-associated fibroblasts. The experiment results demonstrated that AF-SPN provided higher accumulation in tumor tissues than both the uSPN and CC-SPN, and amplified NIR fluorescence and photoacoustic (PA) signals for tumor imaging (Li J. et al., 2018).

## Hybrid Cells Membrane Camouflaged Theranostic Nanocomplex

**FIGURE 5:**
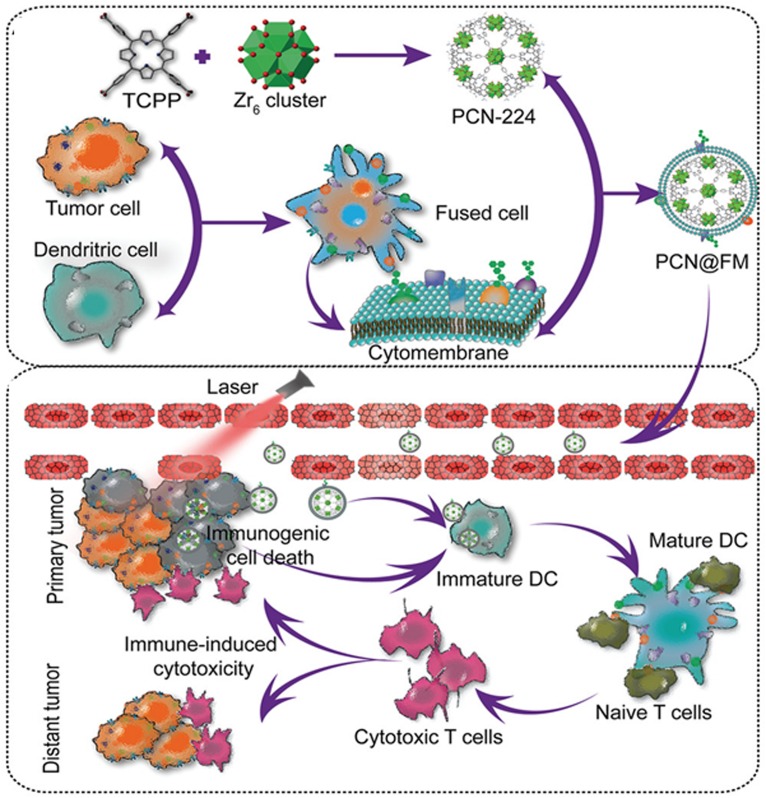
Schematic illustration showed the preparation of PCN@FM for combined tumor therapy. Reproduced with permission from Liu W.L. et al. (2019). Copyright © 2019 WILEY-VCH Verlag GmbH & Co. KGaA, Weinheim.

Based on the concept that membranes from various cell types carry different properties. Research Scientists developed two types of cell membrane fusion coating made nanoparticles inherit and amplify the properties of both source cells (Liang et al., 2018; Ye H. et al., 2019). The two types of pre-extracted cell membranes were mixed together at appropriate protein weight ratios at 37°C to facilitate membrane fusion. For instance, Dehaini et al. fabricated RBC-platelet hybrid membrane-coated nanoparticles. This dual-membrane-coated nanoplatform exhibited long circulation, excellent biocompatibility and suitability for further *in vivo* exploration (Dehaini et al., 2017). In another study, firstly, the researchers get hybrid cells. Briefly, cancerous 4T1 cells and dendritic cells (DCs) were mixed at a ratio of 1:2 in the phosphate buffer (PBS) solution containing 50% polyethylene glycol (PEG) (*MW* = 4000) and 10% dimethyl sulfoxide (DMSO) after 2 min fusion at 38°C, the cells were washed with medium to remove the PEG and DMSO. After fused cells were cultured for 6-day, the cytomembranes (FMs) of hybrid cells were collected. FMs were coated on metal organic framework (PCN-224) by ultrasonic treatment in a cold water bath until the solution was transparent. The obtained hybrid cell membrane coated nanoparticles were further purified by centrifugation to remove the free FMs. The authors showed that this hybrid cell membrane coated nanoparticles can not only inherit the specifically targeted ability to homologous tumors from parent 4T1 cells but also obtained the enhanced ability of immune induction owing to the high expression of a whole array of tumor antigens in FMs (Figure 5; Liu W.L. et al., 2019).

## Other Cells Membrane Camouflaged Theranostic Nanocomplex

Thanks to the advantages of cell membrane coated nanosystem for cancer theranostic, more and more types of cell membrane be used as coating materials according to their self-nature and the clinical need. Platelet, derived from megakaryocytes, is an indispensable component of blood stream, participate in many physiological activities and play an important role, including coagulation, hemostasis, the body’s innate immune response, and cancer metastasis (Li Z. et al., 2018). P-Selectin is a cell adhesion protein, found predominantly in endothelial cells and platelets. Upon platelet activation, it can get exposed on the platelet membrane (PM) surface, and specifically bind to CD44 receptors upregulated on the surface of cancer cells (Bergstrand et al., 2019). Inspired by these properties of platelets, Hu et al. developed a PM coated core-shell nanovehicle (denoted as PM-NV) for codelivery of tumor necrosis factor (TNF)-related apoptosis inducing ligand (TRAIL) and doxorubicin (Dox). The nanocomplex was defined as TRAIL-Dox-PM-NV. TRAIL is one of the most important extracellular activators of apoptosis, induces apoptosis of tumor cells by binding to the death receptors (DR4, DR5) on the cell surface; while Dox can damage the nuclear DNA of cancer cells to trigger the intrinsic apoptosis signaling pathway. PM coating enhanced drug accumulation by active targeting based on the affinity between PM and overexpressed CD44 receptors on the cancer cells. TRAIL-Dox-PM-NV showed synergetic antitumor efficacy to MDA-MB-231 tumor-bearing nude mice (Hu et al., 2015).

At present, the great benefits of immunotherapies in oncology are evident, T cells engineered to express chimeric antigen receptors (CARs) that are specific for tumor antigens have demonstrated tremendous success in eradicating hematologic malignancies (e.g., CD19 CARs in leukemias) (Herzyk and Haggerty, 2018; Labanieh et al., 2018). However, this success was not observed in solid tumors, and the reasons for this are being investigated (Newick et al., 2017). Considering these situations, Ma et al. combined cell membrane coating nanotechnology with CAR-T therapy to treat hepatocellular carcinoma (HCC), due to the high tumor specificity of CAR-T cells and the advantage of cell membrane-camouflaged nanoparticles in drug delivery. They used Glypican-3 (GPC3) targeting CAR-T to prepare CAR-T membranes (CMs). GPC3, a 580-AA heparin sulfate proteoglycan, is a key biomarker for early diagnosis of HCC due to its overexpression in 75% of HCC samples, but not in healthy liver or other normal tissues (Dargel et al., 2015; Zhang Q. et al., 2018). Near-infrared (NIR) dye IR780, was loaded in mesoporous silica nanoparticles (MSNs) to form a core. The IR780 dye with NIR absorbance can produce heat under laser for PTT. IR780-loaded MSNs (IMs) were coated with a layer of pre-prepared CAR-T membranes using an extrusion method to fabricate tumor specific CAR-T Cell membrane-coated nanoparticles (CIMs). CIMs inherited the tumor targeting and a long circulation ability from the membrane cloaking, and demonstrated enhanced anti-tumor capabilities with minimal systematic toxicity both *in vitro* and *vivo* (Ma et al., 2020b).

## Conclusion and Perspectives

This review has highlighted the current development of cell membrane coated nanoparticles for cancer theranostic. We present the overview of the application of RBC membrane coating materials, cancer cell membrane materials, stem cell membrane materials, and others on cancer theranostic. Cell membrane coated nanoparticles have shown unique advantages to enhance cancer therapy and imaging, but they still have many problems need to be overcome in translating to the clinic. For example, the yield of cell membrane extraction is low, it often needs to culture a huge number of cells, and just harvest a small amount of cell membrane, therefore cell isolation and purification approach still requires future improvement. There are various proteins are present on the cell membrane. It also needs to identify the potential proteins and remove unwanted proteins. Although a large number of cell membrane coated nanoparticles have been developed for the integration of cancer diagnosis and treatment, how many of their specific functions have been developed, and whether they have realized the functions envisioned by researchers, are the urgent problems need to be proven. All in all, there is the need to establish standard protocols for obtaining and testing cell membrane coating production. However, the current evaluation of the therapeutic and diagnostic effect of cell membrane coated nanoparticles have been limited in preclinical studies. we hope that the clinical translation of cell membrane coated nanoparticles can be accelerated, which will make a positive impact on human health and be of great economic value.

## Author Contributions

TL contributed to the design, reorganize the figures, and writing the manuscript. XQ, YL, and XS contributed to research the literature. SL, HY, CW, CZ, JZ, and FY helped with editing the manuscript. YL conceived and designed the outline of this review. All the authors read and approved the final manuscript.

## Conflict of Interest

The authors declare that the research was conducted in the absence of any commercial or financial relationships that could be construed as a potential conflict of interest.
